# Secure PUF-Based Authentication Systems

**DOI:** 10.3390/s24165295

**Published:** 2024-08-15

**Authors:** Naing Win Tun, Masahiro Mambo

**Affiliations:** 1 Division of Electrical Engineering and Computer Science, Graduate School of Natural Science and Technology, Kanazawa University, Kanazawa 920-1192, Ishikawa, Japan; 2 Institute of Science and Engineering, Kanazawa University, Kanazawa 920-1192, Ishikawa, Japan

**Keywords:** Internet of Things, physically unclonable function, challenge–response pair (CRPs), Paillier homomorphic encryption, plaintext equality test

## Abstract

The Internet of Things faces significant security challenges, particularly in device authentication. Traditional methods of PUF-based authentication protocols do not fully address IoT’s unique security needs and resource constraints. Existing solutions like Identity-Based Encryption with Physically Unclonable Functions enhance security but still struggle with protecting data during transmission. We show a new protocol that leverages PUFs for device authentication by utilizing Paillier homomorphic encryption or the plaintext equality test to enhance security. Our approach involves encrypting both the challenge–response pairs (CRPs) using Paillier homomorphic encryption scheme or ElGamal encryption for plaintext equality testing scheme. The verifier does not need access to the plaintext CRPs to ensure that sensitive data remain encrypted at all times and our approach reduces the computational load on IoT devices. The encryption ensures that neither the challenge nor the response can be deciphered by potential adversaries who obtain them during the transmission. The homomorphic property of the Paillier scheme or plaintext equality testing scheme allows a verifier to verify device authenticity without decrypting the CRPs, preserving privacy and reducing the computational load on IoT devices. Such an approach to encrypting both elements of the CRP provides resistance against CRP disclosure, machine learning attacks, and impersonation attacks. We validate the scheme through security analysis against various attacks and evaluate its performance by analyzing the computational overhead and the communication overhead. Comparison of average computational and communication time demonstrates Paillier scheme achieves approximately 99% reduction while the plaintext equality test achieves approximately 94% reduction between them.

## 1. Introduction

IoT devices are becoming increasingly popular across various organizations, including in smart homes, healthcare, and offices. However, their widespread use has led to significant security concerns. These devices have risks of impersonation, tampering, and data loss, primarily due to their reliance on wireless connectivity and Internet access, which exposes them to a wide range of cyber threats. Key issues include ensuring proper authentication, authorization, data integrity, confidentiality, and privacy. Addressing these challenges is crucial for leveraging the full potential of IoT technologies and enhancing their security and performance [1]. IoT devices often have limited processing power, a small memory, and low energy supplies, making it hard to use standard authentication methods that need more resources. Traditional security methods, like using public or private key systems, keep secret keys or important data in the temporary memory of devices. This configuration makes them susceptible to physical threats such as invasive, semi-invasive, or side-channel attacks [2]. An attacker could potentially extract the secret key or replicate the entire IoT system, using this to impersonate the device. Therefore, physical attacks pose a significant risk, especially since IoT devices are commonly placed in public spaces where they are easily accessible. To address these issues, PUFs provide a hardware-based security solution through a challenge–response mechanism, generating a unique response to each challenge. This feature makes them a promising tool for enhancing IoT device security. Although physically unclonable functions (PUFs) enhance IoT device authentication—which is vital for safeguarding against unauthorized network access—they remain vulnerable to security threats. Devices and networks still face risks from physical attacks, where attackers can remotely eavesdrop or tamper with network connections [3]. Thus, it is essential to design security measures that are efficient and robust against a range of attacks. The challenges associated with IoT security have spurred the development of a lightweight authentication technique fixed to the unique needs and limitations of these devices. Any authentication method deployed must be robust, computationally efficient, and resilient against physical intrusions. PUFs [2] have emerged as a favored approach for enhancing security affordably. This makes them an attractive choice for creating more secure and affordable authentication methods for IoT environments. However, current PUF-based authentication systems still face significant challenges, including vulnerability to machine learning attacks, CRP disclosure, and the need for secure data transmission. To address these challenges, we propose a novel secure PUF-based authentication protocol that leverages Paillier homomorphic encryption or plaintext equality testing to enhance security. The necessity for the new scheme arises from the current limitations and vulnerabilities of existing PUF-based authentication systems. We validate the scheme through comprehensive security analysis against various attacks and evaluate its performance by analyzing the computational and communication overhead. By addressing these critical security challenges, our protocol aims to provide a robust, efficient, and secure authentication solution for IoT devices, enabling safer and more reliable IoT applications.

### 1.1. Contributions

The integration of the Paillier homomorphic encryption or plaintext equality test with physically unclonable functions-based device authentication in IoT systems significantly contributes to enhancing the security, privacy, and overall integrity of the IoT system. To the best of the authors’ knowledge, there is no such scheme combining PUFs with homomorphic encryption or plaintext equality test, even though the construction looks straightforward. The combination of the unique hardware-based security features of PUFs and data protection capabilities of homomorphic encryption and plaintext equality test addresses several critical challenges in IoT security.

The contributions of this paper are summarized as follows:

- In the authentication process by a PUF IoT device to a gateway router, the correctness of PUF responses can be verified by the gateway router in an encrypted form without any decryption. Processing data in an encrypted form minimizes the risks of data breaches, enhancing trust.
- Under this construction, the resistance against CRP disclosure, machine learning attack, and impersonation attack is improved over the scheme using plaintext CRPs.
- Integrating the PUFs with Paillier encryption or plaintext equality test for device authentication ensures data confidentiality and protection against unauthorized access or tampering.
- Eliminating the storage of secrets on the IoT devices makes the protocol secure against physical attacks.
- The performance and security of the constructed scheme are evaluated to validate the effectiveness of the construction.

### 1.2. Paper Organization

Our paper is organized as follows. First, our contribution is introduced in Section 1. The preliminaries are expressed in Section 2. The related work is described in Section 3. The secure PUF-based authentication protocol is explained in Section 4. Section 5 and Section 6 present a security consideration of the scheme. Then, we discuss the performance evaluation in Section 7 and a conclusion is drawn in Section 8.

## 2. Preliminaries

This section introduces the preliminaries’ background describing the characteristics of physically unclonable function, homomorphic encryption, Paillier encryption, ElGamal encryption and its plaintext equality test using trapdoor.

### 2.1. Physically Unclonable Function

Physically unclonable functions *PUFs* are becoming essential in the hardware security environment. PUFs leverage the natural and small inconsistencies that arise during the semiconductor manufacturing process to create distinct, device-specific signatures or responses. These inconsistencies are unpredictable and nearly impossible to duplicate, positioning PUFs as a choice for authentication, identification, and key generation within secure systems. The fundamental concept of PUFs revolves around their capability to produce a unique response to a specific challenge, determined by the individual physical traits of the device. This challenge–response behavior functions similarly to a cryptographic hash function but is rooted in the hardware’s physical attributes [2]. In IoT systems, PUFs offer a robust mechanism for device authentication and secure key storage without the need for non-volatile memory to store cryptographic keys. This capability is particularly advantageous for IoT devices, which often operate in unsecured environments and are susceptible to physical tampering. By leveraging the unique physical characteristics of devices, PUFs offer a powerful tool for authentication, key generation, and security that is deeply rooted in the hardware itself. One critical function is device authentication, where PUFs ensure that only authorized devices can access networks or services, thwarting impersonation attacks.

**Figure 1:**
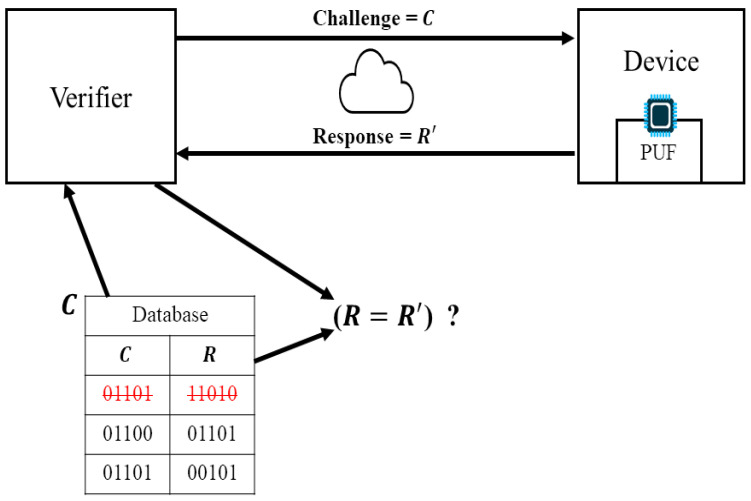
Overview of PUF-based authentication protocol in IoT.

Figure 1 describes the workflow of the PUF-based authentication protocol in an IoT environment. The verifier initiates the authentication process by generating a challenge *C* and sending it to the IoT device equipped with a PUF. The PUF device receives the challenge *C* and uses its unique physical characteristics to generate a corresponding response $R^{\prime }$, which is then sent back to the verifier. The database stores the challenge–response pairs (CRPs) consisting of challenges *C* and their corresponding responses *R*. The verifier retrieves the response *R* from the database and compares it with $R^{\prime }$. If they match, the device is authenticated.

Moreover, leveraging PUFs comes with challenges. Environmental factors such as temperature fluctuations and aging can affect PUF responses, necessitating error correction mechanisms and stable operating conditions to maintain consistency. Sophisticated attackers may employ modeling attacks, attempting to build mathematical models of PUF behavior based on observed challenge–response pairs [4]. To mitigate this risk, the careful management of these pairs and potentially limiting their usage is crucial for robust security. As IoT networks continue to expand and face increasingly sophisticated threats, PUFs stand out as a critical technology in the development of secure, trustworthy IoT systems.

In a PUF experiment, the observed response values can be organized in several ways, highlighting three key aspects and also describing the following [2]:

- Responses obtained from various PUF devices;
- Responses generated by the same PUF device in reaction to different challenges;
- Responses produced by the same PUF device to the same challenge, but during separate evaluations.

### 2.2. Typical PUF-Based Authentication Protocol

**Figure 2:**
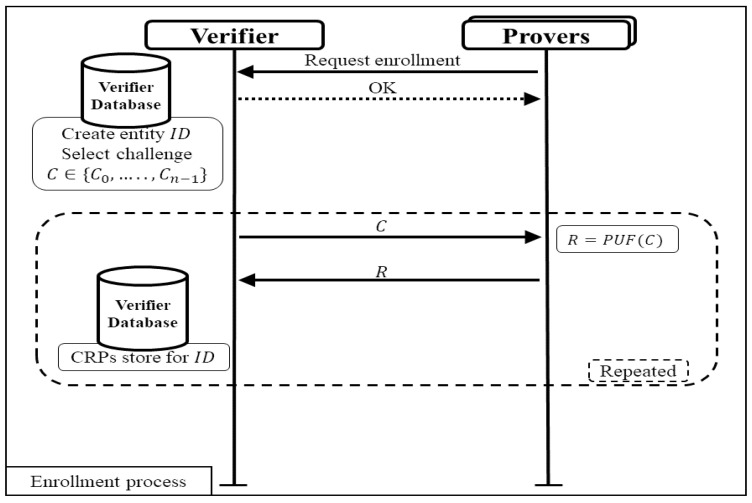
Enrollment process of typical authentication protocol.

A challenge–response protocol using PUFs consists of two processes. This process ensures secure authentication of entities based on their unique PUF characteristics. The first one is the enrollment process that is shown in Figure 2.

In this process, the verifier records the identity of each entity by collecting multiple challenge–response pairs from the PUF of each entity. Various challenges are applied to the PUF, and the corresponding responses are recorded. The collected CRPs are then stored in verifier database, with each entity assigned a unique identifier. The verifier provides each entity with their unique ID for future identification during the authentication process. Each entity can be recognized based on its unique PUF generated responses.

**Figure 3:**
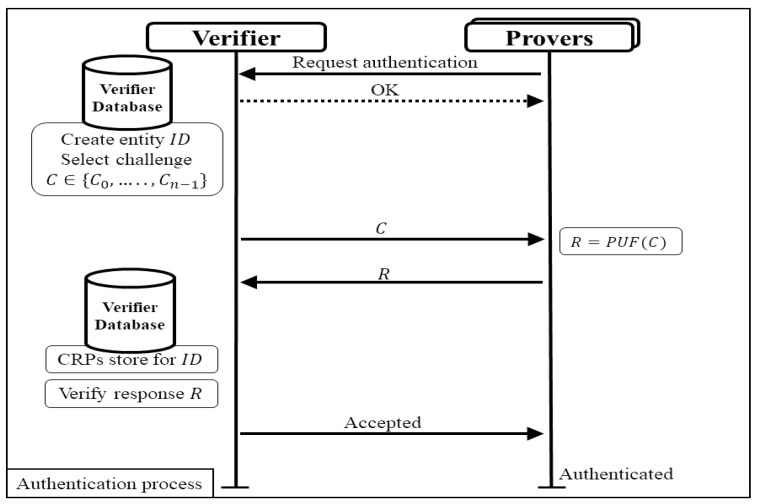
Authentication process of typical authentication protocol.

In the authentication process shown in Figure 3, the entity prover requests authentication by presenting its unique ID to the verifier. The verifier selects the available CRPs associated with the provided ID from the database and randomly selects a challenge. This selected challenge is sent to the prover, who then uses the PUF to generate a response based on the received challenge. The generated response is sent back to the verifier, who compares it with the corresponding response recorded during the enrollment phase. The verifier calculates the distance between the recorded response and the received response for authentication. If the check is successful, the prover is authenticated. The comparison process is vital as it ensures that only genuine responses from the correct PUF can be verified successfully, and it prevents unauthorized access and ensures the reliability of the authentication mechanism.

### 2.3. Encryption Schemes

#### 2.3.1. Homomorphic Encryption

Homomorphic encryption allows computations to be performed on ciphertexts, generating an encrypted result, in decryption, which matches the result of operations performed on the plaintexts which is useful for secure data processing and privacy-preserving computations. The outcome of such computations remains encrypted, ensuring that the process yields the same result as if it had been conducted on unencrypted data. This form of encryption is primarily used to safeguard data privacy when outsourcing data storage and computation, allowing operations on data while keeping these secure. In highly regulated industries like defense and healthcare, homomorphic encryption offers a solution to overcome privacy barriers when sharing data [5,6].

The three main categories of homomorphic encryption include the following:

- Partially homomorphic encryption—PHE;
- Somewhat homomorphic encryption—SHE;
- Fully homomorphic encryption—FHE.

Each of these have varying degrees of computational capabilities on encrypted data.

Additive homomorphism, when given two plaintexts $m_{1}$ and $m_{2}$, and their corresponding ciphertexts $E(m_{1})$ and $E(m_{2})$ under the same public key, will allow [7]:

$$E\left(m_{1},r_{1}\right)⊕E\left(m_{2},r_{2}\right)=E\left(m_{1}+m_{2},r_{1}\times r_{2}\right)$$

where $m_{1}$ and $m_{2}$ are messages, and $r_{1}$ and $r_{2}$ are random numbers associated with the encryption process. This property allows anyone possessing ciphertext to perform an operation between plaintexts, such as summing encrypted values without decrypting them. The security guarantees that, without the corresponding private key, deriving any information about the plaintext from its ciphertext is computationally infeasible. The cryptosystem employs asymmetric key pairs for encryption and decryption, enhancing data security by ensuring that only the holders of the private key can decrypt the information.

#### 2.3.2. Paillier Encryption Scheme

The Paillier cryptosystem [8] is a public-key cryptosystem with the distinctive feature of additive homomorphic encryption. It relies on the mathematical concept of composite degree residuality classes.

*Key generation:* The key generation process involves creating both a public key for encryption and a private key for decryption. It chooses two large prime numbers *p* and *q* such that gcd(pq,(p−1)(q−1))=1. Then, in the computing process, n=pq and λ=lcm(p−1,q−1) where lcm denotes the least common multiple. It selects random integer *g* where $g\in Z_{n^{2}}^{*}$ and compute μ as the modular inverse of $L(g^{\lambda }\;\mathrm{mod}\;n^{2})\;\mathrm{mod}\;n$, where $L\left(u\right)=\frac{u-1}{n}$. (n,g) is public encryption key, and (p,q)(or(λ,μ)) is the private decryption key.
*Encryption process:* To encrypt a plaintext message $m\in Z_{n},$ it chooses a random number $r\in Z_{n}^{*}$ and computes the ciphertext *c* as $c=E\left(m,r\right)=g^{m}\;\cdot \;r^{n}\;\mathrm{mod}\;n^{2}.$
*Decryption process:* To decrypt a ciphertext *c*, it computes $u=c^{\lambda }\;\mathrm{mod}\;n^{2}$. Then, it calculates the plaintext message *m* as $m=L(u)\mu \;\mathrm{mod}\;n=L(c^{\lambda }\;\mathrm{mod}\;n^{2})\mu \;\mathrm{mod}\;n.$

The Paillier scheme finds extensive applications in fields requiring privacy-preserving operations on encrypted data, such as secure voting systems, secure data aggregation in cloud computing [9], and financial privacy, by allowing for encrypted transactions to be aggregated and analyzed anonymously.

#### 2.3.3. ElGamal Encryption and Its Plaintext Equality Test Using Trapdoor

ElGamal encryption is a public-key cryptosystem based on the discrete logarithm problem, which inherently incorporates a trapdoor function. The ElGamal encryption plays a crucial role in its security and functionality [10,11]. The properties of ElGamal encryption in cryptographic communications include the following:

*Setup and key generation:* ElGamal encryption begins with the selection of a large prime number *p* and a generator *g* of the multiplicative group <g> of integers modulo *p*. The private key *x* is chosen randomly from the set $Z_{q}$ where q=|<g>| and the public key *h* is computed as $h=g^{x}\;\mathrm{mod}\;p$. The values *p*, *g* and *h* are publicly shared, while *x* remains secret.
*Encryption process:* To encrypt a message *m*, where *m* is an integer less than *p*, the sender selects a random integer *k* from $Z_{q}$, and computes the ciphertext as a pair $\left(c_{1},c_{2}\right)$ where $c_{1}=g^{k}\;\mathrm{mod}\;p$, $c_{2}=m\times h^{k}\;\mathrm{mod}\;p$.
*Decryption process:* To decrypt the ciphertext $\left(c_{1},c_{2}\right)$, the receiver uses the private key *x* to compute $s=c_{1}^{x}\;\mathrm{mod}\;p$ and obtains $m=c_{2}\times s^{-1}\;\mathrm{mod}\;p$.
*Plaintext equality test using trapdoor:* The paper [10] shows the plaintext equality test of ElGamal ciphertexts using bilinear pairings and a trapdoor which makes efficient and secure equality checks.

For the bilinear mapping, a bilinear map $e:G_{1}\times G_{2}\to G_{T}$ is a function with the following properties:

- Bilinearity: For all $\left(u,\hat{v},a,b\right)\in G_{1}\times G_{2}\times Z_{p}^{2}$, the map satisfies $e\left(u^{a},{\hat{v}}^{b}\right)=e{\left(u,\hat{v}\right)}^{ab}$
- Non-degeneracy: $e(g,\hat{g})\neq 1$, where *g* and $\hat{g}$ are the generators of groups $G_{1}$ and $G_{2}$, respectively.
- Efficient computability: The map *e* can be computed efficiently.

Here, $G_{1}=\langle g\rangle$, $G_{2}=\langle \hat{g}\rangle$, and $\hat{g}\in G_{2}$.

For the trapdoor generation, it is shown as

$$tk=\left(\hat{r},\hat{t}={\hat{r}}^{x}\right)\in G_{2}^{2},\;\mathrm{where}\;\hat{r}\in G_{2}.$$

To compare the two ciphertexts with plaintext equality testing that are as $R=(R_{1},R_{2})$ and $R^{\prime }=\left(R_{1}^{\prime },R_{2}^{\prime }\right)$, encrypt the same plaintext without decrypting them using the following equation involving pairings:

$$e\left(R_{2},\hat{r}\right)\;\cdot \;e{\left(R_{1},\hat{t}\right)}^{-1}=e\left(R_{2}^{\prime },\hat{r}\right)\;\cdot \;e{\left(R_{1}^{\prime },\hat{t}\right)}^{-1}$$

where $R_{1}$, $R_{2}$, $R_{1}^{\prime }$, and $R_{2}^{\prime }$ are elements derived from the ciphertexts. The use of trapdoor elements $\hat{r}$ and $\hat{t}$ derived from the private key *x* ensures that the comparison can be performed without revealing the actual plaintexts, maintaining the confidentiality and integrity of the encrypted data.

## 3. Related Work

Before describing the PUF-based authentication protocols, we present vairous authentication protocols for IoT devices without limiting ourselves only on the PUF-based authentication protocols. The paper [12] introduced a blockchain-based authentication protocol utilizing smart contracts to facilitate the interactions between IoT devices and blockchain smart contracts. However, their approach incorporates the Diffie–Hellman key exchange, which is susceptible to man-in-the-middle attacks. The paper [13] proposed a multifactor authentication protocol that employs PUF and passwords for user verification, exposing it to potential shoulder surfing and denial-of-service (DoS) attacks. The paper [14] designed a mutual authentication framework for the Healthcare IoT sector, where PUF-generated challenge–response pairs are recorded in a database during the registration phase, leaving it open to attacks utilizing machine learning techniques. The paper [15] introduced a protocol that avoids explicitly storing challenge–response pairs in databases by using an offline security credential generator that houses the entire database. Despite its innovation, the protocol also transmits challenges in plaintext, exposing it to potential probing and machine learning attacks through side-channel analysis, as well as denial-of-service attacks. The developed PUF-IPA [16] protocol offers a novel approach to secure thing-to-machine (T2M) authentication. The protocol uses a shuffling method to obscure the storage of physical unclonable functions, enhancing device identity preservation and reducing reliance on conventional challenge–response pairs. This method notably increases resistance to machine learning attacks. However, the PUF-IPA protocol exhibits vulnerabilities, due to insufficient server-side verification of the uniqueness of random numbers, and locking attacks, which exploit the protocol’s response to repeated authentication failures. Liang et al. [17] developed a double PUF-based RFID identity authentication protocol. A significant vulnerability in their protocol is the lack of message authentication, which makes it prone to DoS attacks. The paper [18] provided a similar protocol that, while addressing some security issues, still does not provide anonymity and remains vulnerable to DoS attacks. The paper [19] crafted a lightweight authentication protocol that uses binary string shuffling. This protocol, however, lacks robust defense mechanisms against DoS, and impersonation attacks, rendering it potentially insecure for environments requiring high security. Aman et al. [20] introduced RapidAuth, a PUF-based authentication protocol that incorporates elliptic curve cryptography to enhance resistance to physical attacks. However, the protocol overlooks the critical aspect of noise elimination, which is essential for reliable encryption and decryption processes during authentication. Similarly, Muhal et al. [21] developed a PUF-based authentication protocol where both the device and the server retain an initial session secret key for use during authentication. This storage of sensitive information on the device renders the protocol susceptible to physical attacks. Additionally, their protocol lacks mechanisms for error correction and noise elimination, which are crucial for ensuring the robustness and reliability of the authentication process. The paper [22] introduced a novel device-based authentication protocol that sidesteps traditional error correction methods like fuzzy extractors by employing a deep convolutional neural network. In their approach, a trained model of the device is stored on a trusted server, which later uses this model to verify whether the received response matches the challenge issued to the device. However, this protocol lacks features such as mutual authentication and anonymity and does not address several potential security threats, including modeling, man-in-the-middle, and denial of service *DoS* attacks. In [23], a lightweight PUF-based authentication protocol designed for mutual authentication between IoT devices and a trusted server was developed. The devices are equipped with PUF circuits, while the server maintains a soft model of the IoT PUF. Although the protocol aims to be lightweight, it employs complex computational functions and overlooks critical aspects such as the practical reliability of PUFs and the anonymity of the devices involved.

### 3.1. Review of PUF-Based Authentication Protocols

We classify the following recent PUF-based authentication schemes. The schemes were developed between resource-constrained device and resource powerful devices to establish secure device authentication. Table 1 provides a summary of reviewed PUF-based authentication protocols. We present various types of the attacks in Section 5 and use them for the analysis of schemes of the following subsections. The strength and weakness of the schemes against the attacks are summarized in Table 3 of Section 5.

#### 3.1.1. Secure WiFi Authentication Scheme

Mahalat et al. [24] proposed a PUF-based authentication protocol aiming at securing WiFi authentication for IoT devices. This protocol is adapted for a thing-to-machine authentication scheme, where a WiFi IoT device (prover) is authenticated to a WiFi router (verifier). This serves to enhance the security measures, such as WPA2, by introducing a physical layer of security to counteract the MAC-layer attacks. Each device stores three challenge–responses pairs. The router uses these Challenge-Response Pairs (CRPs) to authenticate the device so that the device is ensured to communicate with the legitimate router processing it’s CRPs. CRPs are updated after each authentication session. The stored CRPs of the registered WiFi devices is in plaintext format in the router database. If this is compromised, the CRPs of the registered WiFi devices will be exposed, enabling the spoofing of the affected devices. Upon receiving an authentication request from the device, the router generates a random nonce and uses the CRPs to compute modified challenges and responses. The WiFi device receives these values, performs computations to extract the nonce, and verifies the router’s authenticity by checking specific response values using its PUF. The device then sends the computed new responses and a hash back to the router. The router authenticates the WiFi device by verifying the responses and then updates the CRPs. However, this protocol relies on the basis of keeping the nonce *n* secret during each authentication session. If an attacker intercepts and records the exchanged messages during an authentication session, they can compute the nonce using the plaintext challenge and the observed modified challenge. Once the nonce is known, the attacker can deduce all original values of the CRPs and predict the new nonce in subsequent sessions, allowing them to spoof the device or router. Although this protocol ensures the physical layer security and mutual authentication, storing CRPs in plaintext at the verifier is insecure. The system can disclose the CRP if the verifier is compromised. Then, this security relies on the secrecy of nonce *n*. If the nonce is discovered, the protocol can compromise the entire authentication process.

#### 3.1.2. Energy Efficient Authentication Scheme

Yanambaka et al. [25] presented an energy efficient authentication protocol for medical IoT devices. The protocol enables a medical device to authenticate itself to a server in a one-way authentication process. The protocol utilizes a classical challenge–response scheme but introduces an obfuscation technique. This protocol addresses the critical issue of identity spoofing in IoMT environments by leveraging physical unclonable functions (PUFs) to generate unique identifiers for devices. In this protocol, the server generates a challenge and computes its response using its embedded PUF. The server then sends this response to the medical device, which uses it as a challenge to produce another response using its own PUF. The device sends this new response back to the server. The server then uses this response as a challenge to produce a final response with its PUF and stores the CRPs’ tuple. Despite these measures, the protocol has several security vulnerabilities. Since the same challenge and response values can be reused in different sessions without incorporating nonces *n*, an attacker can learn the obfuscated CRPs of the device and execute PUF-modeling attacks. This is possible because the challenge and response pairs are sent in plaintext. The attacker can collect the pairs of challenge and response values and build a model of the device’s PUF. If the attacker is an insider, they can disclose the server’s CRPs by tracking the challenges they sent. The protocol is also susceptible to replay attacks due to the freshness indicators in the exchanged messages. This lack of data freshness and source authenticity means that old messages could be reused for impersonation. PUF responses should not be exchanged in plaintext. Eavesdropper intercepts challenge values and their corresponding responses. By intercepting numerous CRPs, the modeling attack becomes feasible. An approach to making the scheme secure is to hash the PUF responses along with nonces, so that the same CRP can be securely reused. Those plaintext CRPs should never be stored in a trusted location in a form that can be directly used for impersonation. While the protocol introduces an efficient method for authenticating medical IoT devices, it also exposes several vulnerabilities. It can address protecting CRPs.

#### 3.1.3. Single CRPs-Based Authentication Scheme

Kim et al. [26] enabled a smart device to be authenticated by a server while mitigating the storage load of CRPs. Instead of storing all CRPs for all registered devices, the server maintains only one CRP per device at any given time. During the registration phase, a single CRP for each device is saved in the database. After each authentication, the stored CRP for a given device is updated. When a device is authenticated, it generates a new CRP and sends it to the server, which then updates its records accordingly. While this approach of the protocol reduces the storage space required for CRPs, it does not entirely eliminate the issue of storing CRPs on the server. After deleting a used CRP, the server maintains a list of challenge values, which grows with the number of authentication attempts. Moreover, the protocol is vulnerable to specific attacks. In this attack scenario, the device initiates authentication by sending its identity to the server. The server responds with a challenge. The device then computes the corresponding response and generates a symmetric key by combining the challenge and response. Then, it sends an encrypted response to the server, which includes a new CRP. The server computes the key, decrypts the message, verifies the response, and if correct, authenticates the device, saves the new CRP, and sends an acknowledgement to the device. Since the authentication of the device is based on successfully responding to a single challenge, an attacker could brute force the response by generating all possible values until an acknowledgement is received from the server. This attack’s feasibility depends on the size of the response. If the size of response is *k*-bits, the attacker would need at most $2^{k}$ attempts. Once the response value is discovered, the secret key is compromised. If the attacker manipulates the authentication response messages to contain invalid CRP values, the server saves the incorrect CRP. This causes all future authentication attempts from the legitimate device to fail until a CRP-updating procedure is initiated to correct the stored CRP. The vulnerability here is that an attacker can brute force the response by trying all possible values until the server acknowledges a correct response. Both the server and the smart device are vulnerable to denial of service (DoS) attacks. For the server, because the authentication messages sent from the device are encrypted, the server must decrypt them before authenticating the source. An attacker could flood the server with bogus messages, forcing it to perform unnecessary decryption operations before discarding the messages. For the device, as the server is not authenticated to it, an attacker could flood the device with spoofed new CRP requests by generating new CRPs. To mitigate the attack, it should use large-sized variables, such as PUF responses, which are difficult to use for an attacker.

#### 3.1.4. CRPs Preserving Authentication Scheme

The protocol in [27], distinguished by its utilization of elliptic curve cryptography operations and a hierarchical architecture for device authentication, represents a significant stride towards enhancing the security of IoT systems. The protocol leverages Identity-Based Encryption with the unique properties of physically unclonable functions *PUF* alongside *ECC*, pairing operations, and hash functions to authenticate IoT devices to verifiers within a hierarchical system. However, the protocol does not protect CRPs during their transmission. The design guarantees that each verifier authenticates a specific subset of IoT devices, with a centralized root security association provider that compiles and maintains CRP data throughout the network. The practical implementation of the protocol was demonstrated using a double arbiter-PUF on a Diligent Nexys-4 FPGA board, showcasing its applicability in real-world scenarios such as video surveillance systems. Despite its innovative approach, the protocol is not resistant to security vulnerabilities. For instance, the countermeasure designed to mitigate denial-of-service attacks—by slowing down or shutting down protocol execution under high request volumes—can paradoxically be exploited by attackers aiming to temporarily incapacitate devices. Moreover, the protocol’s safeguard against repeated requests for the same CRP is susceptible to race-condition attacks, where an attacker spoofs requests in advance, causing legitimate communications to be unjustly rejected by the verifier. A notable concern is the computational demand placed on IoT devices, tasked with performing multiple elliptic curve operations and hash functions for authentication. The requirement not only strains the limited resources of such devices but also presents an avenue for attackers to deplete device power through repeated challenge requests. Suppose an attacker is an insider or an outsider compromising the verifier of authentication protocol. Then such as attacker may use previously accessed authentication data to impersonate devices. All other schemes discussed in Section 3.1.1, Section 3.1.2, Section 3.1.3, Section 3.1.4, Section 3.1.5 and Section 3.1.6 are susceptible of the attack. The protocol’s security assumption hinges on the secure offline storage of CRPs and cryptographic keys within a trusted environment, alongside the use of tamper-proof non-volatile memory (NVM) for storing secret keys. While these measures aim to safeguard critical security parameters, they also underscore the challenges of maintaining such a secure infrastructure, especially considering the cost implications of tamper-proof NVM solutions.

#### 3.1.5. V2G Authentication Scheme

The SUKA protocol introduced in [28] offers an enhancement to the security of electric vehicle *EV* authentication by eliminating the need to store secret keys on the devices themselves, by utilizing physically unclonable functions within smart grid infrastructures. However, vulnerabilities remain for spoofing and message forging attacks due to risks associated with the centralized storage of crucial data by the grid server. Moreover, the initial phase of authentication, which handles vehicle owner identities in plaintext, faces a significant privacy risk. The grid server generates a challenge and computing its response using the server’s PUF. This response is then sent to the EV, which uses it as a challenge to produce another response using its own PUF. The EV sends this new response back to the server. One major concern is the reliance on the secrecy of nonce values used during the authentication process. If an attacker can intercept and analyze these nonce values, they might predict future nonces *N* and compromise the authentication process. The protocol could be susceptible to replay attacks if the nonces are not managed properly. Then, a second vulnerability allows attackers to breach security by cracking the derived session key between the aggregator and the server and using it to reveal all the CRPs of an electric vehicle *EV*. This process can be repeated for each vehicle to disclose its CRPs. The attacker intercepts the messages exchanged during the authentication process. The attacker can decrypt the CRPs of the electric vehicle *EV* cracking the session key. Then, it can enable a spoofing attack before the electric vehicle *EV* performs another authentication session. For attack scenarios, first, if an attacker successfully impersonates the aggregator, they can deceive the electric vehicle *EV*. Second, since the grid server stores the CRPs and identities of all registered electric vehicles in plaintext, a compromised server leads to a complete security breach. Third, the identities of the vehicle owner are transmitted in plaintext during the authentication with the aggregator, making them susceptible to tracking by attackers. CRPs should never be stored in plaintext, regardless of the trust level of the storage location. This can cause CRP disclosure.

#### 3.1.6. Noise-Resilient Authentication Scheme

The protocol introduced by Zerrouki F. et al. [2], known as the fuzzy extractor enhanced authentication protocol, and a mutual authentication and session key establishment protocol tailored for IoT devices utilize the capabilities of silicon PUFs to achieve strong security across IoT devices communication networks. This uniquely generates cryptographic keys from the inherent physical characteristics of IoT devices, integrating a fuzzy extractor to correct errors and produce reliable keys from noisy data sources. Then, it substantiates the protocol’s security and efficiency and comparative performance analyses, demonstrating its superiority in resisting various security threats. The strength of the scheme includes enhanced security through robust defense against physical breaches, minimal computational demand making it ideal for resource-limited IoT devices, and a more secure authentication mechanism compared to traditional cryptographic methods. However, challenges such as the complex implementation requirements due to the precise nature of PUFs, sensitivity to environmental changes affecting reliability, and scalability issues in deploying the system on a large scale are noted as potential drawbacks. Physical attacks pose a significant threat due to the storage of sensitive data on device memory, which could be tampered with to extract cryptographic keys. While the protocol is designed to minimize the risk of modeling attacks by limiting the exposure of challenge–response pairs, it is theoretically possible for sophisticated attackers to use advanced machine learning techniques to develop the models of the PUF from limited data, especially if they can gather enough observations over time. In environments where the server must handle the very high volumes of requests, even with security measures in place, there could still be vulnerabilities to flooding attacks that exhaust computational resources.

#### 3.1.7. Enhanced IoT Security with Geometric Secret Sharing Scheme

The protocol presented in IoT without explicit CRPs in the verifier’s database by K. Nimmy [29] introduces the authentication protocol customized for IoT environments. The protocol leverages physically unclonable functions and a unique geometric threshold secret sharing method to significantly enhance security by eliminating the need to explicitly store challenge–response pairs in a database. This approach not only prevents potential machine learning attacks which exploit CRPs but also reduces the memory requirements, making it ideal for resource-constrained IoT devices. The protocol’s security is rigorously showing robustness against cloning, probing, side-channel, and machine learning attacks. It is described on the prototype of highlighting the protocol’s minimal memory usage and proving its feasibility for real-world applications. This provides a substantial advancement in securing IoT devices, particularly in scenarios prone to physical and remote attacks, setting a new standard for lightweight and secure IoT communications.

## 4. Scheme Overview

### 4.1. IoT Network Architecture

**Figure 4:**
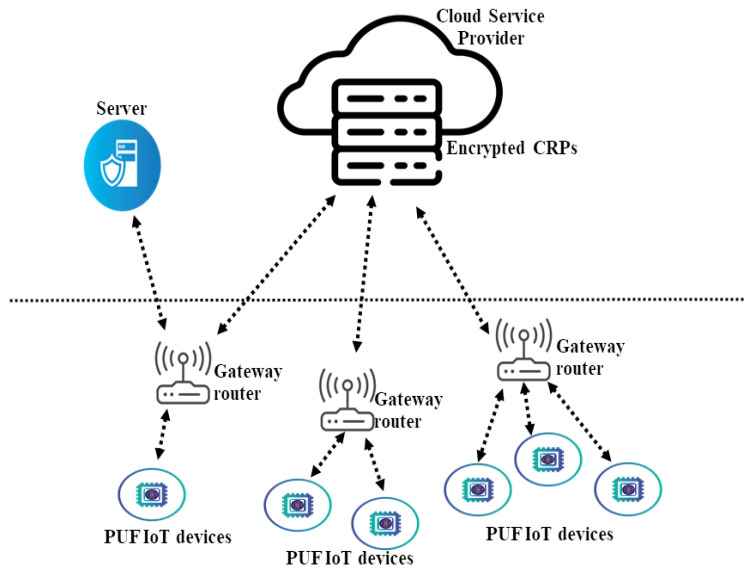
System model.

There are four entities of our scheme in Figure 4, which are as follows.

- PUF IoT devices—*D*: It utilizes physically unclonable functions to generate unique and unpredictable responses based on its inherent physical characteristics. The device acts as the client in the authentication protocol.
- Gateway router—*GR*: It is responsible for initiating challenges, processing responses, and facilitating secure communication. It can also serve as the first layer of authentication and encryption/decryption. The GR can perform initial checks on the encrypted data received from the PUF-IoT devices. Utilizing the properties of Paillier homomorphic encryption or plaintext equality test, the gateway router forwards and retrieves encrypted responses without needing to decrypt them, thus maintaining data confidentiality.
- Cloud service provider—*CSP*: It hosts the cloud storage of encrypted PUF challenge–response pairs *CRPs* for each devices. CSP provides advanced data-processing capabilities, a suitable amount of CRP storage and additional authentication services. This facilitates data retrieval but does not participate directly in the authentication process and guarantees data integrity and availability by implementing comprehensive backup and disaster recovery strategies.
- Server—*TTP*: Trusted third party *TTP* is a critical component in the secure PUF-based authentication protocol and acts as an independent verifier. It is responsible for verifying the equality of encrypted responses from the PUF devices without decrypting the actual responses.

Our secure PUF-based authentication protocol integrates the theoretical functions to enhance the security and efficiency of the network architecture with Paillier homomorphic encryption and privacy preservation and plaintext equality testing. The Paillier encryption scheme allows mathematical operations to be performed directly on ciphertexts. Within the IoT network architecture, the verifier/gateway router can process and verify encrypted challenge–response pairs (CRPs) without the need to decrypt them. This capability preserves the privacy and confidentiality of the CRPs during transmission and verification. The Paillier encryption scheme’s homomorphic properties enable secure computations, such as addition, on encrypted data, facilitating privacy-preserving authentication. For the plaintext equality testing, it provides the ability to compare encrypted values to determine equality without decryption. This allows the verifier/gateway router to confirm whether the encrypted response $R^{\prime }$ from the PUF IoT device matches with the stored encrypted response *R* in the database of cloud service provider.

### 4.2. Assumption

We set the following system assumptions:

- PUF IoT device that is a unique resource-constrained device, and consistently generates the same response when provided with the same challenge under similar environmental conditions.
- PUF IoT devices are secure against physical tampering up to a certain degree of sophistication by attackers.
- PUF IoT devices communicate with the gateway router through the secure channel.
- The server is considered a trusted third party with no resource limitations.
- Cloud service provider securely stores CRPs and has sufficient resources.

### 4.3. Adversary Model

We assume that the adversary model follows the Dolev–Yao (DY) model [30], which is commonly used to demonstrate the security of cryptographic protocols.

- Adversary can eavesdrops on public communication networks.
- Adversary can alter the messages during the authentication process.
- Adversary can forge messages.

### 4.4. Secure PUF-Based Authentication

In our authentication scheme tailored for Internet of Things environments, we leverage the unique capabilities of physically unclonable functions and the cryptographic robustness provided by two distinct schemes: **Scheme-I** (Appendix A) Paillier homomorphic encryption; and **Scheme-II** (Appendix B) plaintext equality testing using trapdoor. These two schemes are the integration of PUFs within IoT devices to generate device-specific cryptographic keys, thereby enhancing the inherent security of device authentication processes. Combining the utilization of the physical unclonable function with Paillier homomorphic encryption or plaintext equality testing facilitates secure and private communication between devices by allowing operations to be performed on encrypted data, ensuring that sensitive information remains protected throughout the authentication process. The schemes are designed to enhance security, making it suitable for resource-constrained IoT devices, and provides a detailed analysis of the protocol’s efficiency in terms of computational overhead and communication cost.

PUF devices are registered to gateway routers and the devices can be authenticated. Our protocol has three main phases: the registration phase, authentication phase, and verification phase. At first, in both the registration and authentication phases, the operation flows are similar for both two schemes. In the verification phase, we describe each scheme separately. The symbols and notations of these cryptographic properties are expressed in Table 2. Our approach not only secures the authentication process against a range of potential security threats but also ensures scalability and ease of deployment across diverse IoT systems, thereby addressing some of the most pressing security challenges in IoT networks.

#### 4.4.1. Registration Phase

**Figure 5:**
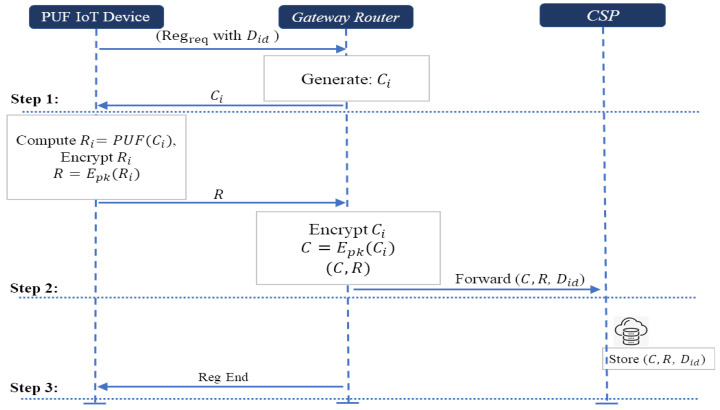
Registration phase.

In the device registration phase for the existing PUF-based authentication protocol [26], most of them store the number of CRPs in the authentication progress to the server database. In our scheme, the gateway router does not store the number of CRPs in its database, and forwards those CRPs to the cloud service provider in order to reduce the security risk by safeguarding the confidentially of storing data and protecting server database resources. In the beginning of operations, the system initializes the public *pk* and private *sk* parameters for those two schemes. The public parameters are generated by the server in **Scheme-I** and gateway router in **Scheme-II**. Let *E(·)* and *D(·)* be an encryption and decryption algorithm of Scheme-I and Scheme-II. The communication between the PUF IoT device and the gateway router is conducted through a secure channel. Note that the secure channel between PUF device and the gateway router does not exist in other phases. Figure 5 illustrates the device registration phase and the steps for conducting the registration phase are listed as follows:

*Step 1. Challenge generation and transmission:* The PUF IoT device requests registration with its device identity $D_{i}d$ to the gateway router. The gateway router generates and sends a random challenge $C_{i}$ to the PUF device.
*Step 2. Response generation and transmission:* After receiving the challenge $C_{i}$ from the gateway router, it computes the response $R_{i}$ as $R_{i}$ = PUF($C_{i}$), and it encrypts that response $R_{i}$ with Paillier homomorphic encryption (**Scheme-I**) or ElGamal encryption (**Scheme-II**). The encrypted response $\bar{R}$ is sent back to the gateway router. The gateway router encrypts the challenge $C_{i}$ with the Paillier homomorphic encryption or ElGamal encryption. Then, it forwards the encrypted challenge and response with PUF device identity $D_{id}$ to the cloud service provider.
*Step 3. Secure storage of CRPs:* The cloud service provider securely stores the encrypted challenge–response pair with PUF IoT device’s identity $D_{id}$, completing the registration process.

For each PUF IoT device, many encrypted CRPs are generated and stored in CSP based on the protocol shown above.

#### 4.4.2. Authentication Phase

**Figure 6:**
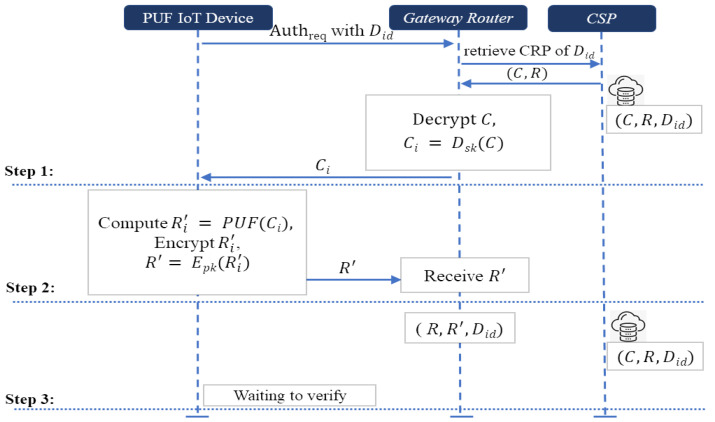
Authentication phase.

The authentication scheme is shown in Figure 6, where we exploit the capabilities of physically unclonable functions and the cryptographic robustness provided by Paillier homomorphic encryption or the plaintext equality testing. The processes between PUF device, gateway router, and cloud service provider are run as follows.

*Step 1. Challenge retrieval and transmission:* The authentication phase begins when the PUF device sends a request containing its unique device identifier $D_{id}$ to the gateway router. Upon receiving the request, the gateway router contacts the cloud service provider to retrieve the encrypted challenge and response associated with the device’s identity. The cloud service provider stores encrypted challenge–response pairs for each registered device. Once the gateway router retrieves the encrypted challenge–response corresponding to the PUF device identity $D_{id}$, it decrypts the encrypted challenge. Then, it sends $C_{i}$ to PUF device.
*Step 2. Response generation and transmission:* The PUF device receives $C_{i}$. Then, it computes the response $R_{i}^{\prime }$, which reacts to the challenge $C_{i}$. It encrypts the response and sends its encrypted response $R^{\prime }$. In this process, the PUF device encrypts the response using Paillier homomorphic encryption in **Scheme-I** or plaintext equality testing in **Scheme-II**.
*Step 3. Retrieving the encrypted response:* To verify the device without decrypting the encrypted responses, the gateway router receives the encrypted response. After that, the gateway router receives two encrypted responses, one of which one is from the PUF device and another which is from the cloud service provider. Note that step 3 of the authentication phase can be executed in the beginning of the verification phase. Finally, the PUF device waits to be verify from the gateway router.

#### 4.4.3. Verification Phase

**Figure 7:**
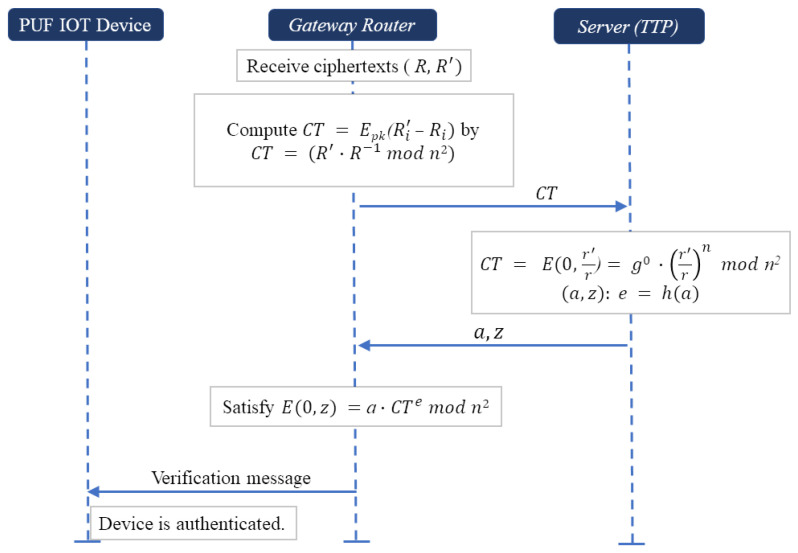
Verification phase utilizing Paillier homomorphic encryption.

*Scheme-I. Utilizing Paillier homomorphic encryption* [31]: A scheme mentioned by Yehuda Lindell is designed to authenticate devices without the need to directly decrypt encrypted responses, as depicted in Figure 7, thereby preserving confidentiality and minimizing the exposure of sensitive data. In the scheme, keys and encryption operations are defined as follows.
*(1) Setup:* The gateway router and the server coordinate to establish the necessary cryptographic parameters:
  - *Key generation:* The server generates the Paillier public and private keys. Let the public key pk=(n,g) where *n* is a multiplication of large numbers *p* and *q*, and *g* is a generator in $Z_{n^{2}}^{*}$. The private key sk=(p,q) or λ=(p−1)(q−1) is kept secret by the server.
  - *Public key distribution:* The public key pk is distributed to the gateway router and potentially to other devices within the network that require encryption capabilities. A cryptographic hash function *h* is shared between the gateway router and the server.
*(2) Encryption phase:* The responses $R_{i}$ and ${Ri}^{\prime }$ are, respectively, encrypted into *R* and $R^{\prime }$ using Paillier encryption $E_{pk}\left(.\right)$ as follows:
  $$R=E_{pk}\left(R_{i}\right)=g^{R_{i}}\;\cdot \;r^{n}\;\mathrm{mod}\;n^{2},$$
  $$R^{\prime }=E_{pk}\left({Ri}^{\prime }\right)=g^{{Ri}^{\prime }}\;\cdot \;{\left(r^{\prime }\right)}^{n}\;\mathrm{mod}\;n^{2}.$$

During the verification phase, the gateway router in the possession of *R* and $R^{\prime }$ performs as follows. The gateway router computes

$$CT=R^{\prime }\;\cdot \;R^{-1}\;\mathrm{mod}\;n^{2}=E_{pk}\left({Ri}^{\prime }\right)\;\cdot \;E_{pk}{\left(R_{i}\right)}^{-1}\;\mathrm{mod}\;n^{2}.$$

Due to the homomorphic properties of Paillier encryption, we obtain:

(1) $$CT=g^{{Ri}^{\prime }-R_{i}}\;\cdot \;{\left(\frac{r^{\prime }}{r}\right)}^{n}\;\mathrm{mod}\;n^{2}.$$

If ${Ri}^{\prime }$ = $R_{i}$, then CT is a ciphertext of zero, i.e.,

(2) $$CT=E\left(0,\frac{r^{\prime }}{r}\right)=g^{0}{\left(\frac{r^{\prime }}{r}\right)}^{n}\;\mathrm{mod}\;n^{2}.$$

The gateway router sends CT to the server.

*(3) Proof and verification* [32]: The server proves to the gateway router the correctness of Equation (2) using its secret key sk, while both the gateway router and the server take (n,CT) as their input.

The server possessing sk can decrypt CT and obtain 0 as its plaintext. The server can further compute $v=\sqrt[{n}]{CT}\;\mathrm{mod}\;n$ where $CT=g^{0}{\left(\frac{r^{\prime }}{r}\right)}^{n}\;\mathrm{mod}\;n.$

The server chooses a random number $\hat{r}\in Z_{n}^{*}$, and computes

$$a=E\left(0,\hat{r}\right)=g^{0}{\hat{r}}^{n}\;\mathrm{mod}\;n^{2}.$$

After computing the value e=h(a), the server computes $z=\hat{r}\;\cdot \;v^{e}\;\mathrm{mod}\;n^{2}.$ Then, the server sends (a,z) to the router. The router checks whether CT,a,z are relatively prime to *n*. In that case, the router computes e=h(a) using a cryptographic hash function *h* and verifies the following Equation (3):

(3) $$E\left(0,z\right)=a\;\cdot \;CT^{e}\;\mathrm{mod}\;n^{2}.$$

Since $a=E\left(0,\hat{r}\right)=g^{0}\;\cdot \;{\hat{r}}^{n}\;\mathrm{mod}\;n^{2}$, one can derive:

$$a\;\cdot \;CT^{e}\;\mathrm{mod}\;n^{2}=g^{0}\;\cdot \;{\hat{r}}^{n}\;\cdot \;{\left(v^{n}\right)}^{e}\;\mathrm{mod}\;n^{2}=g^{0}\;\cdot \;z^{n}\;\mathrm{mod}\;n^{2},$$

and the Equation (3) is satisfied for CT containing 0 as its plaintext.

Therefore, it confirms that the encryption of ciphertext zero without revealing any plaintext values and authenticity of the PUF device. The protocol ensures that the actual responses remain encrypted throughout the process without directly revealing any sensitive information.

**Figure 8:**
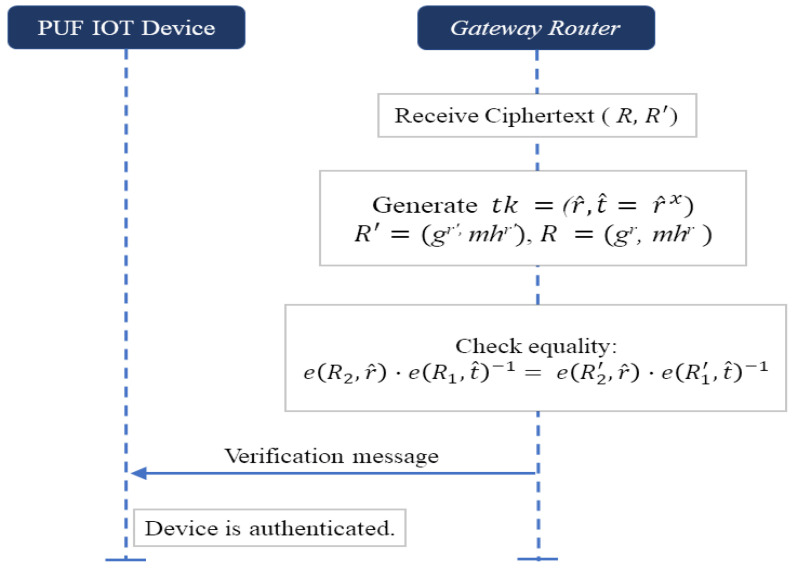
Verification phase utilizing the plaintext equality test.

*Scheme-II. Utilizing Trapdoor for the equality test* [10]: Figure 8 shows a plaintext equality test using trapdoor. This scheme does not involve any server as in Scheme-I. The key and trapdoor generation as well as the receipt of ciphertexts are performed as follows.
*(1) Setup:* The gateway router generates
  $$sk:x\overset{R}{\leftarrow }Z_{p},$$
  $$pk:h=g^{x}\in G_{1},$$
  and the gateway router further generates a trapdoor,
  $$tk=\left(\hat{r},\hat{t}={\hat{r}}^{x}\right)\in G_{2}^{2}$$
  where
  $$\hat{r}\overset{R}{\leftarrow }G_{2}.$$
*(2) Ciphertext receipt:* The gateway router receives ciphertext $R^{\prime }=\left(R_{1}^{\prime },R_{2}^{\prime }\right)=\left(g^{\alpha^{\prime }},m^{\prime }h^{\alpha^{\prime }}\right)$ from the PUF device and ciphertext $R=\left(R_{1},R_{2}\right)=\left(g^{\alpha },mh^{\alpha }\right)$ from the cloud service provider. After receiving ciphertexts, the gateway router can start the plaintext equality test as shown below.
*(3) Plaintext equality test:* The gateway router checks the equality of
  (4) $$e\left(R_{2},\hat{r}\right)\;\cdot \;e{\left(R_{1},\hat{t}\right)}^{-1}=e\left(R_{2}^{\prime },\hat{r}\right)\;\cdot \;e{\left(R_{1}^{\prime },\hat{t}\right)}^{-1}$$
  where *e* is a bilinear map $e:G_{1}\times G_{2}\to G_{T}$ explained in Section 2.3.3. Then, the gateway router starts knowing the equality of plaintexts if the above Equation (4) holds; otherwise, the plaintexts are not equal. The operation determines whether R’ and R are the ciphertexts of the same response value, enabling the comparison of encrypted data without the need for decryption. If the comparison confirms, the device is authenticated. Otherwise, the authentication request is declined.

The phase encapsulates the essential mechanisms ensuring that only legitimate and verified devices can communicate securely through the network, combining PUF technology and the security features of Paillier homomorphic encryption or the plaintext equality test. The proposed methodology not only safeguards against various security threats but also enhances the trustworthiness of device communications within IoT environments.

## 5. Informal Security Analysis

This section evaluates the security of our protocol. The security analysis assesses the protocol’s resilience against known attacks targeting IoT systems, examining its robustness in practical scenarios.

1. *Eavesdropping attack:* Eavesdropping is addressed in our schemes by encrypting the responses $R_{i}$ and $R_{i}^{\prime }$ using public key encryption. The encryption of transmitted data, including challenge–responses pairs CRPs, ensures that even if attackers intercept the data, they cannot access the plaintext responses. Using encryption schemes $E(R_{i})$ ensures data confidentiality. This encryption protects data between the PUF device, the gateway router, and the CSP, preventing unauthorized access to sensitive information.
2. *Man-in-the-middle attacks:* The gateway router verifies the device using encrypted data, preventing the exposure of any decrypted data. Attackers cannot alter or forge legitimate communication. It makes it excessively difficult for attackers to inject or alter messages. Encrypted communication and verification work against this attack.
3. *Denial-of-service (DoS) attack:* The physical unclonability of PUF and encrypted challenge–response pairs reduce the risk of DoS attacks. Encrypted communication allows identify and discard illegitimate requests. While it may not completely prevent DoS attacks, it ensures that the authentication process remains secure against such disruptions.
4. *Machine learning attack:* Employing unique and unpredictable challenges $C_{i}$ and encrypting the responses $R_{i}$ make it difficult for attackers to gather sufficient CRP data to train a machine learning model. Our scheme discards used challenge–response pairs that ensures that the attackers cannot gather a sufficient amount of data are permanently removed. Utilizing Paillier encryption properties or plaintext equality text ensures further security, and reduces the opportunity for attackers to model the behavior of the PUF. Attackers cannot access or decrypt the actual responses from the secure database of the cloud service provider.
5. *CRP Disclosure:* Most of authentication protocols suffer from CRP disclosure attacks and then PUF impersonation [17,33,34]. Our scheme prevents it by encrypting responses $R_{i}$ and $R_{i}^{\prime }$ before transmission and not storing explicit CRPs in the router database. Encrypted storage and transmission ensure that CRPs are never exposed in plaintext. This approach reduces the risk of CRP exposure or unauthorized disclosure.
6. *Replay attack:* An attacker of replay attack intercepts communication and the intercepted data will be reused in later sessions. By performing replay attack in authentication schemes, the attacker can be verified as a legitimate party. In our scheme, the challenge and response pair will not be used in later sessions, and the use of unique and unpredictable responses $R_{i}$ ensures that attackers cannot predict responses to new challenges.
7. *Impersonation attack:* Attackers can attempt to impersonate as a legitimate device using forged PUF responses. However, the unpredictable nature of PUF-generated responses $R_{i}=PUF\left(C_{i}\right)$ ensures that authentication data cannot be replicated or forged. Only the legitimate PUF device can produce the correct response. Even if the attacker obtains discarded CRPs, they cannot derive $C_{i}$ from the encrypted challenge $E_{pk}\left(C_{i}\right)$ without the private key so that the attacker does not know which CRP corresponds to a challenge $C_{i}$ sent from the gateway router.
8. *Physical attack*: PUF is designed to be resistant to cloning and tampering due to their unique and unpredictable response characteristics. Any attempt to physically attack or replicate a PUF device will not yield useful information for generating the correct responses.

Table 3 demonstrates that the secure PUF-based authentication systems are well equipped to handle the spectrum of attacks, ensuring robust security for IoT devices.

## 6. Formal Security Validation: AVISPA Simulation Tool

To perform a rigorous security proof of our protocol, we employ the SPAN+AVISPA toolset [35]. SPAN, the security protocol animator for AVISPA, is an essential tool designed specifically for CAS+ and HLPSL (high-level specification language) specifications on the SPAN-Ubuntu 10.10-light OS. By utilizing SPAN’s active intruder implementation, we are able to interactively identify and model attacks on protocols. AVISPA is instrumental in determining whether a security protocol is resistant to active and passive attacks.

In evaluating protocol security, AVISPA leverages four primary back-end components: the constraint logic-based attack searcher (CL-AtSe), the SAT-based model checker (SATMC), the on-the-fly model checker (OFMC), and the tree automata-based protocol analyzer (TA4SP) [36]. However, due to compatibility issues, AVISPA software is not compatible with the SATMC or TA4SP back-ends. And, we utilize the CL-AtSe and OFMC back-ends to assess the safety of our protocol.

**Figure 9:**
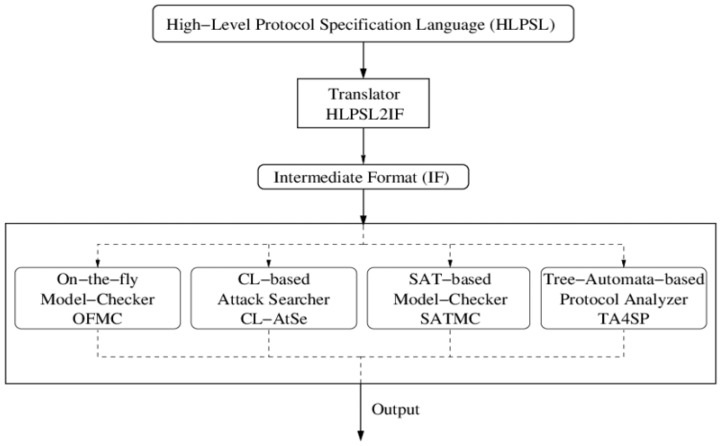
Architecture of the AVISPA tool.

We employ HLPSL to define the protocol, which is then translated into intermediate format (IF) for compatibility with AVISPA, as shown in Figure 9. The IF specifications are subsequently analyzed using model checkers such as OFMC and CL-AtSe. These analyses are conducted under the Dolev–Yao threat model, which simulates an intruder with capabilities of eavesdropping, message alteration, and message forging. Both CL-AtSe and OFMC conduct extensive searches for malicious intruders to ensure that only authorized parties can execute the specified protocol. The entities in the protocol are depicted as fundamental roles. These fundamental roles outline the initial conditions and state transitions. The messages exchanged by the entities throughout both phases are transmitted and received via the SND and RCV channels. The information about regular sessions between legitimate entities is then provided to the intrusive party by these back-ends.

### Formal Security Analysis

To ensure that our protocol is robust against replay, man-in-the-middle, impersonation attack, and CRP disclosure, we utilized the AVISPA simulation tool, which operates on the Dolev–Yao model. AVISPA uses the high-level protocol specification language (HLPSL) to validate the security of protocols through detailed role specifications.

We describe it using rule-based HLPSL, setting up various roles for the IoT device, the gateway router, and the server, as well as defining the security goals, environment, and sessions. We then check the security of paillier encryption scheme **Scheme-I** using the OFMC and CL-AtSe model checkers. Upon executing the HLPSL scripts within AVISPA, our protocol assessed that its evaluation is “SAFE”. This indicates that the suggested protocol exhibits strong protection against the Dolev–Yao threat model, showing resilience against such as CRP disclosure, impersonation, replay and Man-in-the-Middle (MITM) attacks.

We focused on the OFMC and CL-AtSe back-ends. Our secure PUF-based authentication protocol includes four primary roles: PUF device, gateway router, and trusted third party-server, and cloud service provider. The simulation also considers the intruder as an active participant in the protocol execution.

**Figure 10:**
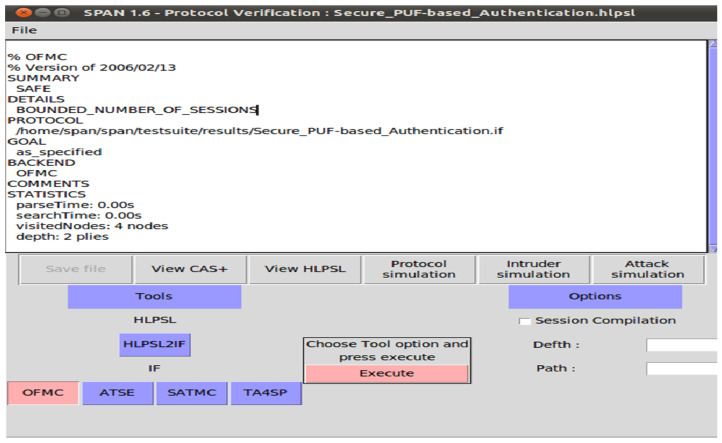
Security analysis simulation result of OFMC.

**Figure 11:**
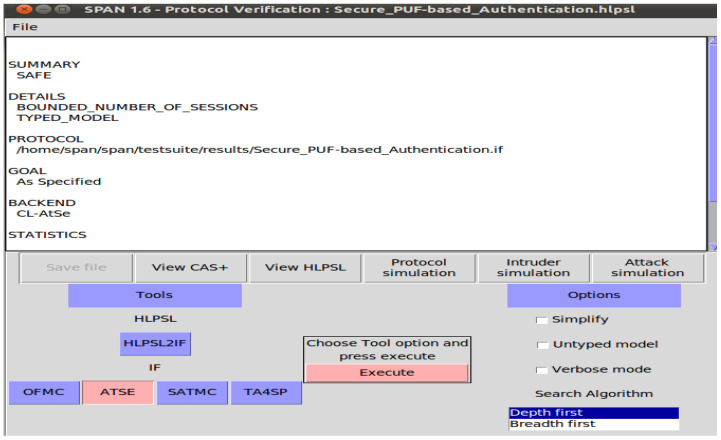
Security analysis simulation result of CL-AtSe.

We conducted simulations to verify the security properties of our protocol. However, we utilized the Security Protocol Animator for AVISPA (SPAN) tool to animate the protocol’s execution and visualize the security analysis. The results are described in Figure 10 and Figure 11 which demonstrate that our protocol provides security guarantees and mitigates various known attacks.

The AVISPA and SPAN tools confirm that our protocol is secure and resilient against such attacks, validating its effectiveness for secure PUF-based communications in IoT environments.

## 7. Performance Evaluation

### 7.1. Computational Overhead

In this section, the efficiency of our schemes stems from the statistical analyses for cryptographic performance. We compare the computational overhead between our schemes.

We implement our protocol using cryptographic libraries to compare between Scheme-I and Scheme-II. The experiments are conducted in a controlled software environment. To compute the communication overhead, the experiments are run on the Windows 11 machine with Intel(R) XEON(R) W-1250 CPU @3.30GHz (12 CPUs) and 32GB of Memory that are deploying on VMs with 8CPU on 8GB of Memory. The bit length of each of challenge and response is set to be 128 in parameter settings.

Table 4 describes the approximate computational time of each cryptographic operations utilized in encryption libraries such as GNU multiple precision arithmetic (GMP) [37] and pairing-based cryptography (PBC) [38] for large integer arithmetic. We employ cryptographic libraries [39] to handle bilinear pairing generation and cryptographic operations. Those schemes are executed using the parameters derived from a pairing with a 224-bit group order at the specified 112 bits security level [40] and a bit length of security parameter n=2048 is used. To simulate the operations, we run the operation process 100 times repeatedly to analyze the average approximate time of the computational performance, which is detailed in Table 4. Table 5 shows a comparison of the computational costs between the two schemes. It should be noted that the verification phase of **Scheme-I** in Table 5, Table 6 and Table 7 do not count for the computations of decryption of CT as well as the $n^{th}$ root of CT, i.e., the random number $\left(\frac{r^{\prime }}{r}\right)\;\mathrm{mod}\;n$ inside CT.

Table 5, Table 6 and Table 7 describe the computational costs during the registration, authentication, and verification phases, highlighting the impact of cryptographic operations on the overall performance. The performance efficiency of two schemes have differences in computational costs. **Scheme-II** has an additional computational overhead during the verification phase to compute pairing operations, which are computationally expensive. In contrast, **Scheme-I** is more efficient in computational costs without pairing operations. $T_{\left(PUF\right)}$ denotes the time associated with PUF operations in Table 5. It is involved in the registration and authentication phases.

Table 6 describes the computational costs of cryptographic operations of two schemes. It does not include the time for PUF computation. According to [41], the response time of PUF computation in general is 0.02 ms. In Table 7, the computational costs with the PUF computation of two schemes is described.

We evaluate how much time increases by the cryptographic operation of our schemes in comparison with the PUF-based authentication schemes without any cryptographic operation. The computational cost of the latter authentication scheme without cryptographic operation is 0.0608 ms. It is the sum of time for the random number generation time 0.0001 ms, the PUF generation time 0.02 ms, computational time 0.0003 ms of comparing two strings in verification. The cryptographic operation in Scheme-I increases the time by 0.0012 ms, while in Scheme-II, it increases the time by 13.5375 ms compared to the PUF-based authentication schemes without any cryptographic operation. **Scheme-I** avoids the computationally expensive pairing operations, making it more efficient in terms of computational overhead. **Scheme-II**, while offering potentially stronger security due to pairing operations, incurs a higher computational cost in the verification phase. From the viewpoint of IoT device’s computational cost, **Scheme-I** and **Scheme-II** have similar encryption process. Both schemes maintain their low energy consumption on the IoT devices, ensuring that the energy efficiency is preserved.

### 7.2. Communication Overhead

To examine the analysis of performance evaluation, we analyze the communication performance of two schemes concerning communication costs in terms of the number of message transmissions and the total bits transmitted.

**Scheme-I** involves the interaction with a server during the verification phase, resulting in additional message exchanges between the gateway router and the server. The total number of message transmissions is 11 messages and 45,056 bits, which is more than the number of messages and higher in bits compared to the 2688 bits transmitted in **Scheme-II**. The interaction ensures that the verification of the encrypted zero is handled securely without direct decryption, enhancing security at the cost of increased communication overhead and latency.

**Scheme-I** eliminates the need for interaction with a server. The verification of plaintext equality is managed directly by the gateway router using plaintext equality tests using pairing operations. The absence of a server simplifies the protocol, reducing potential latency and complexity associated with involving an additional party.

The following Table 8 and Table 9 describe the communication overhead analytically for both schemes.

**Scheme-I** involves a more extensive communication process, with three message exchanges during the registration phase, four during the authentication phase, and another four during the verification phase, summing up to a total of 11 message exchanges and 45,056 bits transmitted. This scheme requires an interaction with the server during the verification phase, which ensures the secure handling of the encrypted zero. While this enhances the security of the protocol, it comes at the cost of increased communication overhead and latency.

On the other hand, **Scheme-II** is designed to be more efficient, with only two message exchanges during the authentication phases, and a single message exchange during the verification phase. This results in a total of 6 messages exchanged and 2688 bits transmitted. By avoiding server interaction, this scheme simplifies the protocol, reducing potential latency and complexity while maintaining the integrity of the verification process through the use of pairing operations. The average communication time highlighted in **Scheme-II** achieves approximately 94% reduction compared to **Scheme-I**.

Comparing the two schemes, Scheme-I prioritizes security with its additional server interaction with a higher communication overhead and increased latency. In contrast, Scheme-II achieves greater efficiency by minimizing the number of messages and bits transmitted, directly handling verification at the gateway router, reducing both latency and complexity.

**Figure 12:**
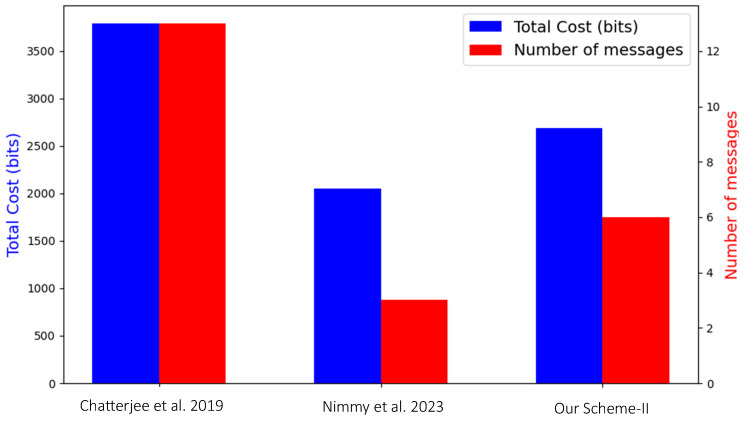
Communication overhead [27,29].

To compare and compute the communication overhead with other existing schemes, Figure 12 provides a side-by-side comparison of the total cost in bits and the number of messages for the selected schemes. The total bits and number of messages for the schemes is shown in Figure 12.

The paper [27] demonstrates a high total communication cost of approximately 3792 bits and requires 13 messages. This scheme exhibits the highest communication overhead among the evaluated schemes.

In [29], a reduced communication overhead is shown with a total cost of around 2049 bits and 7 messages.

Our **Scheme-II** exhibits the lowest communication overhead with a total communication cost of approximately 1500 bits and requiring only 3 messages. This significant reduction in both metrics highlights the efficiency of Scheme-II. **Scheme-I** is not included in the Figure 12 because it demonstrates a significantly higher communication overhead due to the use of the server (TTP) involvement.

## 8. Concluding Remarks

We presented a secure PUF-based authentication system to avoid challenge–response disclosure, and to avoid key storage in Non-Volatile Memory (NVM), which mitigates potential attacks. Our PUF-based authentication scheme utilizes PUFs to enhance the security and preserve privacy more effectively. **Scheme-I** provides strong security guarantees through homomorphic properties and robust verification via a server (TTP), involving higher communication costs due to the involvement of an additional party. In computational costs, **Scheme-I** is more efficient than **Scheme-II**. On the other hand, **Scheme-II** employs computationally heavy pairing operations for plaintext equality tests achieves lower communication costs due to the absence of a server. Our protocol has certain limitations, while our schemes perform efficiently for small-to-medium-sized IoT networks. Scalability to larger networks with thousands of devices is limited by increased communication and computational overhead. Server interaction in **Scheme-I** introduces latency, impacting real-time applications. Additionally, our security analysis relies on both the robustness of the underlying cryptographic primitives and the PUF’s resistance to physical attacks. These limitations need to be overcome by enhancing scalability to support large-scale IoT deployments, e.g., by integrating blockchain technology and by assessing the impact of quantum computing on the underlying cryptographic primitives and PUF’s resistance. 

## Figures and Tables

**Table 1:** Summary of review in authentication protocols.

Protocols	Scheme	Year	Possible Attacks
Mahalat et al. [24]	Secure WiFi	2018	CD, DoS
Yanambaka et al. [25]	Energy efficient	2019	CD, ML, I
Kim et al. [26]	Single CRP	2019	CD, DoS, I
Chatterjee et al. [27]	CRPs preserved	2019	CD, ML, I
Bansal et al. [28]	V2G	2020	CD, I
Zerrouki et al. [2]	Noise resilient	2023	CD, ML, I
Nimmy et al. [29]	Secret sharing	2023	CD

CD—CRP disclosure; ML—machine learning; I—impersonation; DoS—denial of service.

**Table 2:** Notations used in our scheme.

Notations	Definitions
$D_{id}$	Device’s identity
$C_{i}$	Challenge generated for PUF
$R_{i}$	Response generated for PUF
pk	Public key
sk	Secret key
GR	Gateway router
D	PUF device
CSP	Cloud service provider
TTP	Trusted third party (server)
PUF(·)	Physical unclonable function
$Reg_{req}$	Registration request
$Auth_{req}$	Authentication request
CRPs	Challenge–response pairs
tk	Trapdoor
$D_{sk}$	Decryption with private key

**Table 3:** Attack resistance comparison related with PUF-based authentication protocols.

Against Attacks	[24]	[25]	[26]	[27]	[28]	[2]	[29]	Ours
Eavesdropping	Y	N	Y	Y	Y	Y	Y	Y
MiTM attack	Y	N	NM	Y	Y	Y	Y	Y
DoS attack	N	NM	N	NM	NM	NM	Y	Y
Machine learning	N	N	NM	N	NM	N	Y	Y
CRP disclosure	N	N	N	N	N	N	NM	Y
Impersonation	N	N	N	N	N	N	Y	Y
Replay	N	N	NM	Y	Y	Y	Y	Y
Physical	Y	Y	Y	Y	Y	Y	Y	Y
Security proof	UN	UN	UN	M, E	M	S	S, E	S, E

N—no; Y—yes; NM—not mentioned; S—simulation; UN—unspecified; E—experiment; M—mathematical.

**Table 4:** Approximate time (ms) of cryptographic operations.

Symbols	Operations	Scheme-I	Scheme-II
T*_(ME)_*	Modular exponentiation	0.0023	0.0100
T*_(MM)_*	Modular multiplication	0.0005	0.0026
T*_(R)_*	Random number generation	0.0001	0.0001
T*_(MI)_*	Modular inverse	0.0031	0.0008
T*_(P)_*	Pairing computation	-	3.3675

**Table 5:** Comparison of computational overhead of our schemes.

Schemes	Registration	Authentication	Verification
Scheme-I	4T*_(ME)_*+T*_(PUF)_*+	3T*_(ME)_*+2T*_(MM)_*+	T*_(MM)_*+T*_(MI)_*
	2T*_(MM)_*+2T*_(R)_*	T*_(R)_*+T*_(PUF)_*	
Scheme-II	4T*_(ME)_*+T*_(PUF)_*+	3T*_(ME)_*+2T*_(MM)_*+	4T*_(P)_*+2T*_(MM)_*+
	2T*_(MM)_*+2T*_(R)_*	T*_(R)_*+T*_(MI)_*+T*_(PUF)_*	2T*_(MI)_*

**Table 6:** Computational overhead of cryptographic operations of our schemes.

Schemes	Registration	Authentication	Verification	Total Times (ms)
Scheme-I	≈0.0104	≈0.008	≈0.0036	≈0.0220
Scheme-II	≈0.0454	≈0.0361	≈13.4768	≈13.5583

**Table 7:** Computational overhead of our schemes with PUF computation.

Schemes	Registration	Authentication	Verification	Total Times (ms)
Scheme-I	≈0.0304	≈0.0280	≈0.0036	≈0.0620
Scheme-II	≈0.0654	≈0.0561	≈13.4768	≈13.5983

**Table 8:** Communication overhead comparison.

Metrics	Scheme-I	Scheme-II
Number of messages	11	6
Total bits transmit	45,056 bits	2688 bits

**Table 9:** Detailed communication overhead.

Phases	Metric	Scheme-I	Scheme-II
Registration	Messages	3	3
	Bits	12,288 bits	1344 bits
Authentication	Messages	4	2
	Bits	16,384 bits	896 bits
Verification	Messages	4	1
	Bits	16,384 bits	448 bits

**Figure A1:**
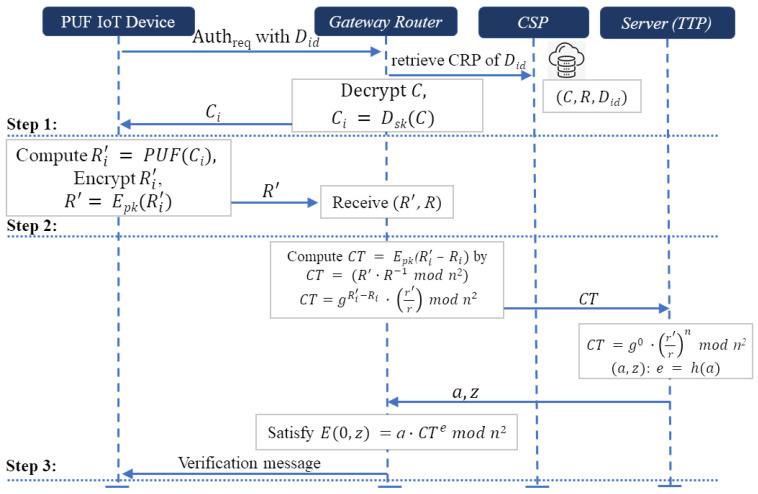
Integrating PUF-based authentication Scheme-I.

**Figure A2:**
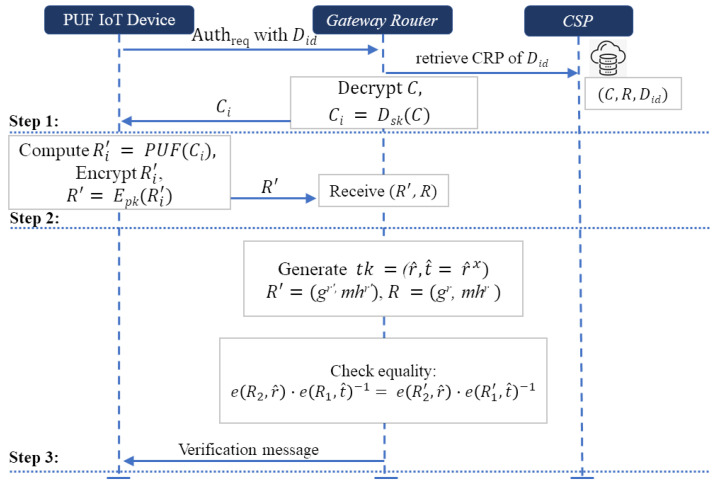
Integrating PUF-based authentication Scheme-II.

## Data Availability

No new data were created or analyzed in this study. Data sharing is not applicable to this article.

## Abbreviations

The following abbreviations are used in this manuscript:

PUF	Physically Unclonable Functions
PHE	Paillier Homomorphic Encryption
CRPs	Challenge–Response Pairs
GR	Gateway Router
CSP	Cloud Service Provider
TTP	Trusted Third Party
NVM	Non-Volatile Memory

## Appendix A. Integrating PUF-Based Authentication Scheme-I

The system combines as a complete procedure to form a comprehensive and secure PUF-based authentication protocol. The system leverages the unique capabilities of physically unclonable functions (PUFs) and the cryptographic robustness of Paillier homomorphic encryption for Scheme-I. We present an integrated procedure of our scheme. Before the authentication and verification phases can be executed, each PUF-based IoT device needs to register through a registration process. This ensures that the device is uniquely identified and securely integrated into the system. This registration process establishes a secure foundation for the subsequent authentication and verification phases, ensuring that each PUF IoT device is uniquely and securely identified within the system.

As illustrated in Figure A1, the individual steps of the authentication and verification phases are combined into a single comprehensive diagram integrating secure PUF-based authentication systems. Appendix A.1 describes with in detail the process of the complete procedure of Scheme-I.

### Appendix A.1. Procedure Utilizing Paillier Homomorphic Encryption Scheme-I

- *Step 1.1:* The PUF device sends an authentication request containing its unique device identifier $D_{id}$ to the gateway router.
- *Step 1.2:* The gateway router contacts the CSP to retrieve the encrypted challenge and response associated with the device’s identity $D_{id}$.
- *Step 1.3:* The CSP stores encrypted challenge–response pairs for each registered device. The gateway router retrieves the encrypted challenge–response corresponding to the PUF device identity $D_{id}$ and decrypts the challenge.
- *Step 1.4:* The decrypted challenge $C_{i}$ is sent to the PUF device.

- *Step 2.1:* The PUF device receives $C_{i}$ and computes the response $R_{i}^{\prime }=PUF\left(C_{i}\right)$.
- *Step 2.2:* The PUF device encrypts the response using Paillier homomorphic encryption in Scheme-I, resulting in $R^{\prime }=E_{pk}\left(R_{i}^{\prime }\right)$.
- *Step 2.3:* The encrypted response $R^{\prime }$ is sent back to the gateway router.
- *Step 2.4:* The gateway router receives the encrypted response $R^{\prime }$.
- *Step 2.5:* The gateway router receives two encrypted responses—one from the PUF device and another from the CSP.
- *Step 3.1:* The gateway router computes $CT=R^{\prime }\;\cdot \;R^{-1}\;\mathrm{mod}\;n^{2}=E_{pk}\left(R_{i}^{\prime }\right)\;\cdot \;E_{pk}{\left(R_{i}\right)}^{-1}\;\mathrm{mod}\;n^{2}$.
- *Step 3.2:* Due to the homomorphic properties of Paillier encryption, the gateway router calculates $CT=g^{R_{i}^{\prime }-R_{i}}\;\cdot \;{\left(\frac{r^{\prime }}{r}\right)}^{n}\;\mathrm{mod}\;n^{2}$.
- *Step 3.3:* If $R_{i}^{\prime }=R_{i}$, then CT is a ciphertext of zero: $CT=E\left(0,\frac{r^{\prime }}{r}\right)=g^{0}\;\cdot \;{\left(\frac{r^{\prime }}{r}\right)}^{n}\;\mathrm{mod}\;n^{2}$.
- *Step 3.4:* The gateway router sends CT to the Server for verification.
- *Step 3.5:* The server, using its private key sk, decrypts CT to verify that it is indeed a ciphertext of zero.
- *Step 3.6:* The server computes $v=\sqrt[{n}]{CT}\;\mathrm{mod}\;n$ and chooses a random number $\hat{r}\in Z_{n}^{*}$, computing $a=E\left(0,\hat{r}\right)=g^{0}{\hat{r}}^{n}\;\mathrm{mod}\;n^{2}$.
- *Step 3.7:* The server computes the value e=h(a) using a cryptographic hash function *h*, and then computes $z=\hat{r}\;\cdot \;v^{e}\;\mathrm{mod}\;n^{2}$.
- *Step 3.8:* The server sends (a,z) to the gateway router.
- *Step 3.9:* The gateway router verifies whether CT,a,z are relatively prime to *n*. If they are, the router computes e=h(a) and verifies Equation $E\left(0,z\right)=a\;\cdot \;CT^{e}\;\mathrm{mod}\;n^{2}$.

The entire procedure from the initial authentication request to the final verification is clearly outlined, ensuring a secure and efficient process for the PUF-based authentication system utilizing Paillier homomorphic Scheme-1.

## Appendix B. Integrating PUF-Based Authentication Scheme-II

For **Scheme-II**, we combine as a complete procedure to form a secure PUF-based authentication protocol. The system leverages the unique capabilities of physically unclonable functions (PUFs) and the cryptographic robustness of the plaintext equality test.

The Figure A2 and Appendix B.1 describe the individual steps of the authentication and verification phases combined into a single comprehensive diagram integrating secure PUF-based authentication systems detailing the process of the complete procedure of Scheme-II.

### Appendix B.1. Procedure Utilizing Plaintext Equality Testing Scheme-II

- *Step 1.1:* The PUF device sends an authentication request containing its unique device identifier $D_{id}$ to the gateway router.
- *Step 1.2:* Upon receiving the request, the gateway router contacts the CSP to retrieve the encrypted challenge and response associated with the device’s identity $D_{id}$.
- *Step 1.3:* The CSP stores encrypted challenge–response pairs for each registered device. The gateway router retrieves the encrypted challenge–response corresponding to the PUF device identity $D_{id}$ and decrypts the challenge.
- *Step 1.4:* The decrypted challenge $C_{i}$ is transmitted to the PUF device.
- *Step 2.1:* The PUF device receives $C_{i}$ and computes the response $R_{i}^{\prime }=PUF\left(C_{i}\right)$.
- *Step 2.2:* The PUF device encrypts the response using plaintext equality testing of the ElGamal encryption in Scheme-I, resulting in $R^{\prime }=E_{pk}\left(R_{i}^{\prime }\right)$.
- *Step 2.3:* The encrypted response $R^{\prime }$ is sent back to the gateway router.
- *Step 2.4:* The gateway router receives the encrypted response $R^{\prime }$.
- *Step 2.5:* The gateway router receives two encrypted responses: one from the PUF device and another from the CSP.
- *Step 3.1:* The gateway router generates the private key sk and public key pk: $sk:x\overset{R}{\leftarrow }Z_{p},$
  $$pk:h=g^{x}\in G_{1},$$
  - The gateway router further generates a trapdoor: $tk=\left(\hat{r},\hat{t}={\hat{r}}^{x}\right)\in G_{2}^{2}$ where $\hat{r}\overset{R}{\leftarrow }G_{2}$.
- *Step 3.2:* The gateway router receives the ciphertext $R^{\prime }=\left(R_{1}^{\prime },R_{2}^{\prime }\right)=\left(g^{\alpha^{\prime }},m^{\prime }h^{\alpha^{\prime }}\right)$ from the PUF device and ciphertext $R=\left(R_{1},R_{2}\right)=\left(g^{\alpha },mh^{\alpha }\right)$ from the CSP.
- *Step 3.3:* The Gateway Router checks the equality of:
  $$e\left(R_{2},\hat{r}\right)\;\cdot \;e{\left(R_{1},\hat{t}\right)}^{-1}=e\left(R_{2}^{\prime },\hat{r}\right)\;\cdot \;e{\left(R_{1}^{\prime },\hat{t}\right)}^{-1}$$

If the equation holds, the plaintexts are equal, and the device is authenticated. Otherwise, the authentication request is declined.
